# Optimisation of quantitative lung SPECT applied to mild COPD: a software phantom simulation study

**DOI:** 10.1186/s13550-015-0086-2

**Published:** 2015-03-22

**Authors:** Pernilla Norberg, Anna Olsson, Gudrun Alm Carlsson, Michael Sandborg, Agnetha Gustafsson

**Affiliations:** Medical Radiation Physics, Department of Medical and Health Sciences, Linköping University, Linköping, 581 83 Sweden; Center for Medical Image Science and Visualization (CMIV), Linköping University, Linköping, 581 83 Sweden; Clinical Physiology, Department of Medical and Health Sciences, Linköping University, Linköping, 581 83 Sweden; Department of Medical Physics, Karolinska University Hospital, Huddinge, Stockholm 141 86 Sweden

**Keywords:** SPECT, Quantitative evaluation, Lung diseases, Computer-assisted image analysis, Technegas, Simulation

## Abstract

**Background:**

The amount of inhomogeneities in a ^99m^Tc Technegas single-photon emission computed tomography (SPECT) lung image, caused by reduced ventilation in lung regions affected by chronic obstructive pulmonary disease (COPD), is correlated to disease advancement. A quantitative analysis method, the CV_T_ method, measuring these inhomogeneities was proposed in earlier work. To detect mild COPD, which is a difficult task, optimised parameter values are needed.

**Methods:**

In this work, the CV_T_ method was optimised with respect to the parameter values of acquisition, reconstruction and analysis. The ordered subset expectation maximisation (OSEM) algorithm was used for reconstructing the lung SPECT images. As a first step towards clinical application of the CV_T_ method in detecting mild COPD, this study was based on simulated SPECT images of an advanced anthropomorphic lung software phantom including respiratory and cardiac motion, where the mild COPD lung had an overall ventilation reduction of 5%.

**Results:**

The best separation between healthy and mild COPD lung images as determined using the CV_T_ measure of ventilation inhomogeneity and 125 MBq ^99m^Tc was obtained using a low-energy high-resolution collimator (LEHR) and a power 6 Butterworth post-filter with a cutoff frequency of 0.6 to 0.7 cm^−1^. Sixty-four reconstruction updates and a small kernel size should be used when the whole lung is analysed, and for the reduced lung a greater number of updates and a larger kernel size are needed.

**Conclusions:**

A LEHR collimator and 125 ^99m^Tc MBq together with an optimal combination of cutoff frequency, number of updates and kernel size, gave the best result. Suboptimal selections of either cutoff frequency, number of updates and kernel size will reduce the imaging system’s ability to detect mild COPD in the lung phantom.

## Background

Obstructed airways and parenchymal destruction are characteristics of chronic obstructive pulmonary disease (COPD). Varying degrees of abnormalities are typically found in different parts of the COPD lung, and some parts of the lung may even be normal. Therefore, the first abnormality to be detected in the early stages of the disease is abnormal ventilation distribution [1]. Since the lung behaves in such an irregular manner, these regional differences in ventilation are preferably studied with the use of single-photon emission computed tomography (SPECT) imaging [2]. Early detection of lung function reduction is important in order to prevent further lung degeneration.

We have previously presented the quantitative CV_T_ method [3,4] which measures inhomogeneities in lung SPECT images. The purpose of the CV_T_ method is to discriminate between activity distributions in the lungs of healthy subjects and subjects with mild COPD. The method has been reported in detail previously. Briefly, the coefficient of variation (CV = the standard deviation divided by the mean) is calculated for overlapping cubic volumes covering the 3D reconstructed activity distribution. The CV values are plotted as a density curve with an area under the curve (AUC) of 100%. A diseased lung is characterised by larger proportion of high CV values compared to a healthy lung. The proportion of high CV values increases with disease advancement (increased heterogeneity of the activity distribution). A CV threshold value (CV_T_) has been determined as the most frequently occurring CV value in the distributions of healthy subjects. Finally, the AUC(CV_T_), the area under the density curve (AUC) for CV values greater than CV_T_, is calculated for both healthy subjects and subjects with COPD. The method has been shown to be capable of identifying simulated mild COPD in an anthropomorphic software phantom (sensitivity = 95%, specificity = 90%) and patients with severe COPD (sensitivity = 100%, specificity = 100%). The parameter values used in that study [3] were not optimised but were good enough for the purpose of demonstrating the usefulness of the suggested method. Since identifying mild lung function reduction is a very difficult task for the SPECT system in a clinical context, it is important that the parameter values used are optimised.

In the literature, a range of lung SPECT imaging parameter values are used [3,5-9] depending on the different tasks addressed. For example, optimal parameter values for qualitative analysis might not be optimal for quantitative analysis. To our knowledge, only Palmer et al. has reported an optimisation procedure and that is for qualitative analysis. Guidance in finding the optimal parameter values can be obtained using trade-off plots of image quality parameters such as contrast, noise and resolution [10-12] or using normalised mean square errors [13,14], but none of these strategies are suitable for optimising the parameter values for the CV_T_ method. The optimal parameter values have to be found in relation to the images used in the CV_T_ method and its quantitative task. The images' visual appearance is therefore of secondary importance.

The ultimate test of the applicability of the CV_T_ method will require access to a large group of well-defined healthy subjects and a large group of well-defined patients with mild COPD. Lacking access to these groups of subjects, Monte Carlo simulated lung SPECT images of software phantom lungs will be used.

The aim of this study was to determine, when using the CV_T_ method, which parameter values of acquisition, reconstruction and analysis maximise the separation between the activity distributions in the lungs of healthy subjects and in the lungs of subjects with mild COPD (small heterogeneity variations).

## Methods

### The software lung phantom and Monte Carlo simulations

The NCAT phantom [15] with a lung volume (air, blood and parenchyma) of 4.2 l was used. The lung volume corresponded to a 65-year-old male (70 kg, 180 cm) in a supine position and in the middle of the respiratory cycle [16,17]. The arms were held over the head. The respiratory motion and heartbeat of the phantom were activated throughout the study, i.e. the phantom was dynamic. The phantom consisted of a 256 × 256 × 256 matrix with a voxel size of 0.165 × 0.165 × 0.165 cm^3^. The muscle, fat, lung, spine bone, rib bone, blood and heart were the selected tissues. Densities and elemental compositions of these tissues were obtained from ICRP 89 [18] and XCOM photon interaction cross sections from Berger et al. [19].

Two different activity distributions were defined (Table 1). The activity distribution in the lungs of a healthy subject (hereafter called healthy distribution) was represented by a homogeneous distribution throughout the lungs. The activity distribution in the lungs of a subject with mild COPD (hereafter called COPD distribution) was represented by a non-homogeneous distribution modelled as small spherical lesions with a diameter of 1 cm within an otherwise homogenous activity distribution. The activity concentrations in the lesions were 50% of the concentration in the healthy surrounding lung tissue. The reduced activity levels in the lesions reflect the magnitude of the reduced ventilation or perfusion. No activity was located elsewhere in the body. The density of the lesions was the same as for the healthy lung simulating lack of activity beyond narrow and/or closed airways. In Table 1, coronal slices are shown, including motion artefacts, to illustrate the distributions observed by the gamma camera during projection acquisition.

**Table 1:** **Activity distributions and their descriptions**

	**Coronal slice**	**Distribution number and description**
Healthy distribution		1: Homogeneous activity distribution
COPD distribution		2: Lesions with a diameter of 1 cm with 50% activity concentration, evenly distributed over the lung volume, occupying 10% of the total lung volume

Coronal slices include motion artefacts.

SPECT projections from the activity distributions were simulated using the SIMIND software, version 4.9d [20]. The projection data incorporated the effects of non-uniform attenuation and scatter. The isotope ^99m^Tc was used corresponding to the use of Technegas in a clinical setting. The energy window was set between 130 and 154 keV to improve contrast-to-noise ratio [21,22]. A non-circular gamma camera rotation orbit was used, corresponding to the auto-contouring system used in our clinic. The centre of rotation to collimator distance varied between 17 and 25 cm.

Simulations were made for a GE Infinia gamma camera (Milwaukee, WI, USA) (0.95-cm-thick NaI crystal) equipped with a low-energy high-resolution collimator (LEHR) and a low-energy general-purpose collimator (LEGP). Projections were collected at 128 different angles, equally spaced, over 360°, in 128 × 128 matrices (0.33 × 0.33 cm^2^ per detector element). A total of 1.8 × 10^10^ photons were simulated with the homogeneous activity concentration, resulting in projections with low noise levels. The CV of one pixel element within the high count area of the lung in one single projection, determined from three consecutive simulations, was 0.5%.

### Normalisation and statistical noise

Clinically realistic noisy projections were generated by scaling the simulated projections and then replacing the pixel values by random deviates drawn from a Poisson distribution [12,13]. The total number of counts in the simulated projections for both activity distributions was set according to Table 2. These values correspond to activities of 25 and 125 MBq contained in the lungs using LEGP and LEHR collimators, respectively [3]. For each activity distribution, 40 noise realisations were created, simulating 40 SPECT acquisitions of the same activity distribution.

**Table 2:** **The total number of counts in the 128 simulated projections**

	**Administered activity**	
	**25 MBq**	**125 MBq**
LEGP	1.23 × 10^6^	6.14 × 10^6^
LEHR	0.73 × 10^6^	3.64 × 10^6^

### SPECT reconstruction and filtering

Each set of 40 noisy projections was reconstructed using the iterative ordered subset expectation maximisation (OSEM) [23] reconstruction software developed at John Hopkins University, Baltimore, MD, USA. The reconstruction included correction for attenuation, scatter and geometrical collimator detector response (CDR) (also known as point spread function (PSF) reconstruction and resolution recovery). Since it is a simulation study, the phantom attenuation coefficients were known and attenuation correction was performed using the average attenuation coefficient map over the respiratory cycle of the phantom. The scatter correction was performed using effective source scatter estimation (ESSE) [13,24]. An analytic geometrical model for CDR compensation was used for each collimator. The reconstructions were performed using 16 subsets and ten different numbers of iterations (see Table 3). The isotropic voxel size in a reconstructed image was 0.33 × 0.33 × 0.33 cm^3^. The reconstructed images were post-filtered with a Butterworth filter [25] with four different cutoff frequencies and a power of 6 (Equation 1) or not filtered at all.

**Table 3:** **Variables used in the study**

	**Values**
Acquisition	
Activity levels	25 and 125 MBq
Collimators	LEHR and LEGP
Reconstruction	
Number of iterations	2, 4, 6,…, 20 with 16 subsets, i.e. 32 to 320 updates
Cutoff frequencies	0.4, 0.5, 0.6, 0.7 cm^−1^ and no filtering
Analysis	
Kernel edge lengths	1.0, 1.7, 2.3 and 3.0 cm (i.e. 3, 5, 7 and 9 voxels)
Volume of analysis	The whole lung and the reduced lung

1 $$ A(f)={\left\lfloor 1+{\left(\frac{\left|f\right|}{Q}\right)}^p\right\rfloor}^{-0.5} $$

where *A*(*f*) is the amplitude of the filter at a spatial frequency *f* and *Q* is the cutoff frequency that controls roll-off and *p* is the power factor.

### Analysis

The CV_T_ method used for the evaluation generates a global measure of inhomogeneity, the AUC(CV_T_)-value, constructed using a kernel-based CV distribution [3,4]. Four kernel edge lengths were used in the evaluation (see Table 3). Each combination of activity level, collimator, number of updates, cutoff frequency of the noise reduction filter and kernel edge length is here called a design. The total number of designs was 800. For each design and activity distribution (Table 1), 40 density curves of CV values based on the 40 noise realisations were generated. For each design, the density curves of the healthy distribution generated a mean frequency function. Its modal value was then used as the threshold CV_T_ value. Finally, the AUC for CV values greater than CV_T_ (i.e. AUC(CV_T_)) was calculated for the two activity distributions. The same procedure was repeated for each design.

#### Statistic evaluation

For each design and activity distribution (Table 1), 40 AUC(CV_T_) values were obtained from the 40 density curves (described above). The non-parametric Mann–Whitney *U* test was used when comparing results from the two activity distributions (healthy and COPD cases). Since the number of values in each group is 40, i.e. larger than 10, the normal approximation to the Mann–Whitney *U* distribution can be applied and *Z*-statistics and *p* values can be calculated [26]. The Bonferroni correction [27] for multiple comparisons was applied reducing the rejection level of the statistical hypothesis test by a factor of 1/800. In this study, the *p* values were not only used for hypothesis testing but also to rank the designs. The *p* values corresponding to the negative *Z*-statistic values were not included in the ranking. The lowest *p* value corresponds to the best separation between the AUC(CV_T_) values for the healthy and COPD distributions and defines the optimal design.

### Defining lung volumes

The lung voxels in the homogenous distribution (Table 1), containing values greater than half the maximum lung value, were set to represent the segmented lung. A reduced lung volume was made by eroding the outer boundary of the segmented lung by one voxel. Both the whole lung and the reduced lung volumes were evaluated in the final optimisation (see Table 3).

### Image and computer processing

The addition of Poisson noise, post-filtration, AUC(CV_T_) calculation and Mann–Whitney *U* test was performed using in-house software developed in Interactive Data Language (IDL; ITT Visual Information Solutions, Boulder, CO, USA).

## Results

**Figure 1:**
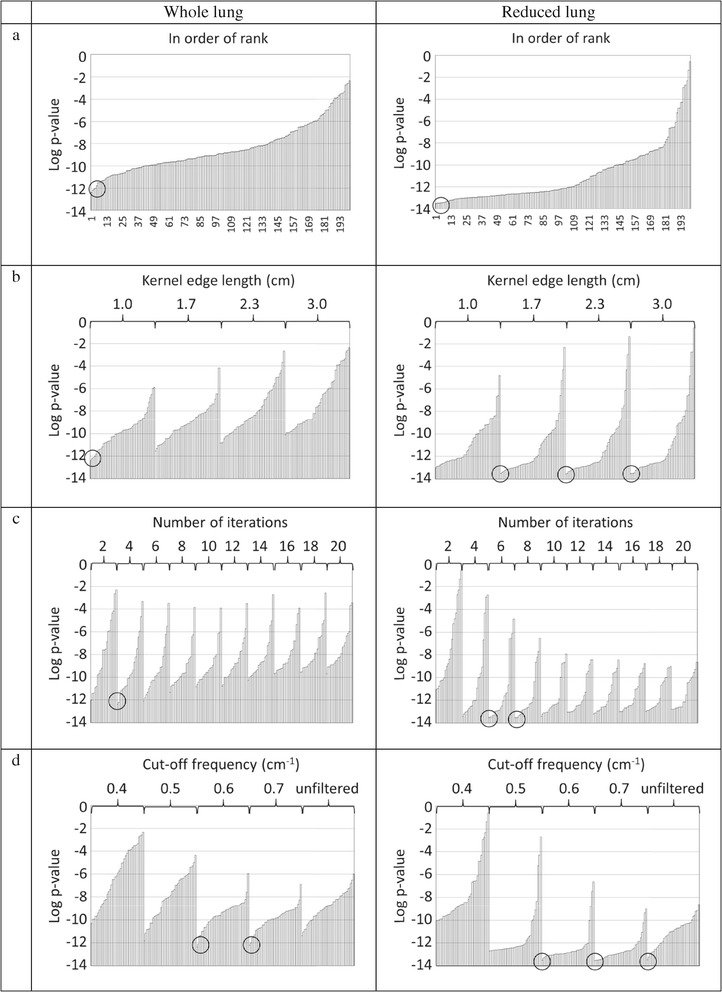
***p*** **values for all the LEHR-125 MBq designs.** Designs for the whole lung are shown in the left column and for the reduced lung in the right column. In row **(a)** the designs are placed in order of rank based on resulting *p* values. In row **(b)** the designs are grouped by kernel size, row **(c)** by iteration number and in row **(d)** by cutoff frequency of the Butterworth filter. The lowest *p* values are encircled.

When the whole lung is analysed, the lowest *p* values result from using LEHR-125 MBq designs. LEHR-125 MBq designs placed in order of rank based on resulting *p* values and thereafter grouped by kernel size show the lowest *p* value for the smallest kernel size, i.e. with an edge length of 1.0 cm (see Figure 1b). When the designs are grouped by number of iterations, a minimum is found at four iterations, i.e. 64 updates (see Figure 1c). When grouped by cutoff frequency of the Butterworth filter, a minimum at 0.6 to 0.7 cm^−1^ is found (see Figure 1d). The lowest *p* value for these designs is *p* = 4.1 × 10^−13^. Designs generating the 10 lowest *p* values are listed in Table 4. The lowest *p* value for a LEGP-125 MBq design is *p* = 8.0 × 10^−4^, a LEGP-25 MBq design *p* = 0.016 and for a LEHR-25 MBq design *p* = 0.020. Also shown in Figure 1 is that a LEHR-125 MBq design can result in a higher *p* value than the best LEGP-125 MBq design, e.g. if a kernel edge length of 3.0 cm, 288 updates and a cutoff frequency of 0.4 cm^−1^ are chosen, *p* = 0.0026 will result.

**Table 4:** **The 10 designs generating the lowest** ***p*** **values for the whole lung**

**Order of rank**	**Kernel edge length (cm)**	**Number of iterations (updates)**	**Cutoff frequency (cm** ^**−1**^ **)**	***p*** **value**
1	1.0	4 (64)	0.6	4.1 × 10^−13^
2	1.0	4 (64)	0.7	6.2 × 10^−13^
3	1.0	6 (96)	0.6	7.9 × 10^−13^
4	1.0	2 (32)	0.7	9.4 × 10^−13^
5	1.0	6 (96)	0.5	1.5 × 10^−12^
6	1.0	6 (96)	0.7	2.8 × 10^−12^
7	1.7	4 (64)	0.7	3.0 × 10^−12^
8	1.0	2 (32)	0.6	3.7 × 10^−12^
9	1.0	2 (32)	Unfiltered	4.2 × 10^−12^
10	1.0	8 (128)	0.6	4.5 × 10^−12^

All designs use LEHR collimator and 125 MBq.

Also, for the reduced lung, the lowest *p* values result from LEHR-125 MBq designs. Low *p* values for LEHR-125 MBq designs are found for the three largest kernel sizes with an edge length of 1.7 to 3.0 cm, six to eight iterations (i.e. 96 to 128 updates) and a cutoff frequency of 0.7 cm^−1^ (see Figure 1). The lowest *p* value for these designs is *p* = 2.8 × 10^−14^. Designs generating the 10 lowest *p* values are listed in Table 5. The lowest *p* value for a LEGP-125 MBq design is *p* = 2.0 × 10^−8^, a LEGP-25 MBq design *p* = 0.0043 and for a LEHR-25 MBq design *p* = 5.2 × 10^−4^. An example of a combination of a LEHR-125 MBq design for the reduced lung with a kernel edge length of 3.0 cm, 32 updates and a cutoff frequency of 0.4 cm^−1^ results in a *p* value as high as 0.26.

**Table 5:** **The 10 designs generating the lowest** ***p*** **values for the reduced lung**

**Order of rank**	**Kernel edge length (cm)**	**No of iterations (updates)**	**Cutoff frequency (cm** ^**−1**^ **)**	***p*** **value**
1	2.3	6 (96)	0.7	2.8 × 10^−14^
2	1.7	8 (128)	0.7	3.1 × 10^−14^
3	2.3	8 (128)	0.6	3.1 × 10^−14^
4	3.0	8 (128)	0.7	3.1 × 10^−14^
5	3.0	6 (96)	0.7	3.3 × 10^−14^
6	3.0	6 (96)	Unfiltered	3.3 × 10^−14^
7	3.0	4 (64)	0.7	3.5 × 10^−14^
8	1.7	10 (160)	0.7	3.8 × 10^−14^
9	2.3	8 (128)	0.7	4.4 × 10^−14^
10	1.7	6 (96)	0.7	4.7 × 10^−14^

All designs use LEHR collimator and 125 MBq.

**Figure 2:**
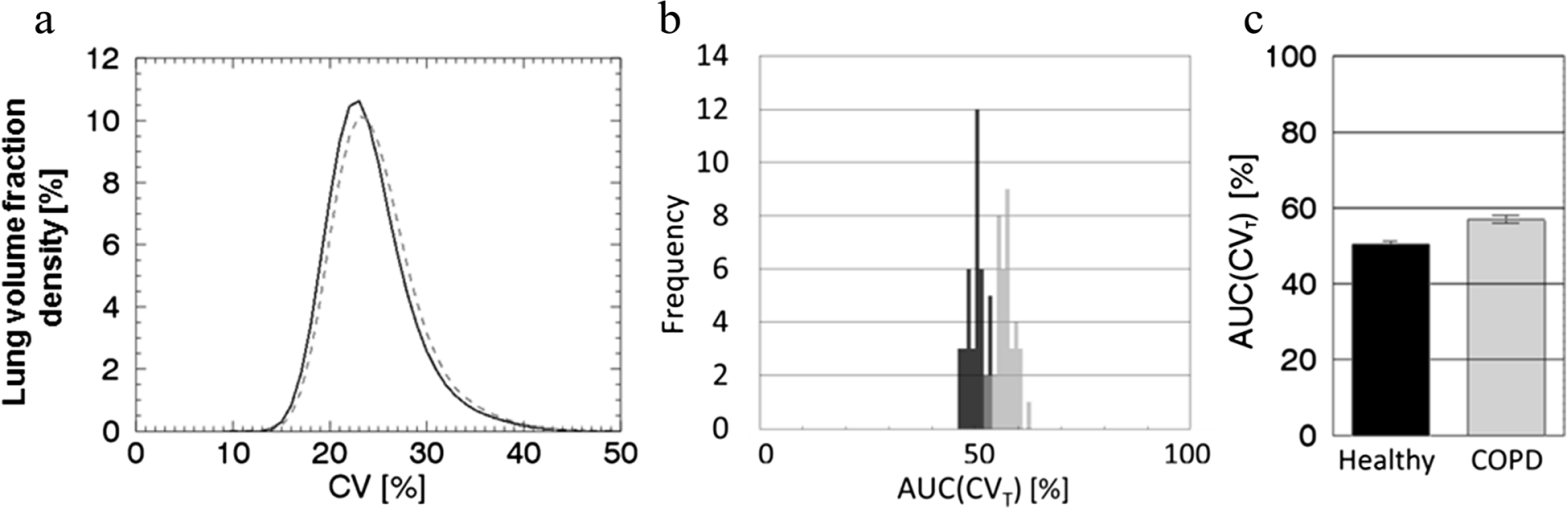
**Mean density curves and AUC(CV**_**T**_ **) distributions. (a)** The mean density curves of the 40 noise realisations of the CV values for the imaged healthy distribution (black line) and imaged COPD activity distribution (grey line) for the top-ranked LEHR-125 MBq design in Table 5 (a kernel edge length of 2.3 cm, six iterations (96 updates) and a cutoff frequency of 0.7 cm^−1^). **(b)** Histograms of the AUC(CV_T_) values of the 40 noise realisations for each activity distribution. **(c)** The same information as in **(b)** but visualised as mean values with 95% confidence intervals. Data are for the reduced lung.

Figure 2 shows the CV mean density curves and AUC(CV_T_) distributions of the healthy and the COPD distributions for the top-ranked LEHR-125 MBq design and a reduced lung. The threshold value (CV_T_) for this design is 23.0%. The mean frequency function of the imaged COPD distribution is shifted towards higher CV values compared to the mean frequency function of the imaged healthy distribution (see Figure 2a). This shift results in higher AUC(CV_T_) values for the COPD distribution compared to the healthy distribution, as shown in Figure 2b,c. For this design, there is no overlap of the two AUC(CV_T_) distributions, and therefore the resulting *p* value is very low (see Table 5).

For the majority of the evaluated designs, the Mann–Whitney *U* test results in positive *Z*-statistic values. A positive *Z*-statistic appears when the AUC(CV_T_) distribution of the imaged COPD distribution appears to the right of the imaged healthy distribution, as in Figure 2b. A negative *Z*-statistic value, on the other hand, appears when their relative positions are reversed. Only positive *Z*-statistic values are generated for the LEHR-125 MBq designs as valid for both lung volumes. Negative *Z*-statistic values instead most frequently appear for the LEGP collimator, low numbers of updates, low cutoff frequencies of the filter and for designs including the whole lung.

## Discussion

In the present study, we have optimised the CV_T_ method with respect to the parameter values of acquisition, reconstruction and analysis in order to maximise the ability of the method to distinguish between images of healthy and mild COPD distributions. We have found that 125 MBq and a LEHR collimator together with a Butterworth post-filter with a cutoff frequency of 0.6 to 0.7 cm^−1^ should be used. When the whole lung was analysed, 64 updates of the OSEM algorithm together with a kernel edge length of 1.0 cm gave the best results. For the reduced lung volume, a larger number of updates and larger kernel sizes were needed.

In our previous work [3], we evaluated the outcome of the CV_T_ method using a variety of different simulated COPD activity distributions. The COPD distribution that was most difficult to separate from the simulated healthy distribution was chosen in this study. The aim was to find the parameter values that gave the CV_T_ method the best chance of succeeding in identifying the smallest lesions. This non-homogeneous activity distribution was created with the aim of mimicking mild COPD where the reduced activity concentration in the small spherical lesions reflects the magnitude of reduced ventilation. The reduction of ventilation of 50% in 10% of the lung volume corresponds to a 5% total reduction of the functioning lung tissue. Compared to the normal range of spirometric variables of about 15% to 20%, we believe this distribution would be very representative of a case of mild COPD. In this distribution, the lesions are evenly distributed throughout the whole lung volume. Grouped lesions have been shown [3] to be easier to resolve compared to evenly distributed lesions and were therefore not chosen in this study.

Since the spatial resolution of the SPECT system using an LEHR collimator is about 1.5 cm (expressed as FWHM at the applied orbit), the COPD activity distribution illustrates activity inhomogeneities that are blurred, resulting in reduced contrast. In the reconstructed images, most of the inhomogeneities are due to an insufficient number of counts and few are due to true inhomogeneities. Despite these difficulties, a separation between the healthy and COPD distribution was found.

Tables 4 and 5 show good combinations of kernel sizes, number of updates and cutoff frequencies for LEHR-125 MBq designs. The differences between the 10 designs listed were small and all of them are good choices. The important message is that even for LEHR-125 MBq designs, a bad combination of the parameter values would reduce the difference between a healthy and a diseased lung (Figure 1). All values have to be properly selected. One wrongly chosen parameter value can worsen the ability of the resulting system dramatically. An example, for the reduced lung, is LEHR-125 MBq together with a kernel edge length of 3.0 cm and 64 updates. Changing the cutoff frequency from 0.7 to 0.4 cm^−1^ increased the *p* value from 3.5 × 10^−14^ to 0.0019.

Using the optimal parameter values obtained in this work improved the ability of the CV_T_ method to distinguish between mild COPD and healthy distributions compared to previously published results [3]. The optimisation reduced the resulting *p* value from 5.8 × 10^−13^ to 2.8 × 10^−14^ for the COPD distribution and the reduced lung volume.

For quantitative assessment of small and less distinct lesions, as in this study, the image needed a high-count density and a high spatial resolution. The LEGP collimator could not resolve the small lesions sufficiently for either of the two activity levels. The study also showed that 25 MBq together with the LEHR collimator had an excessively low count level. Increasing the number of counts increased the separation between the imaged healthy and COPD distributions. Therefore, in a clinical setting, it is important to reach a higher level of inhaled activity, which may be facilitated by coaching in breathing. To increase the separation further than shown in this study, even higher count levels than 3.64 × 10^6^ are needed, which can be achieved by increasing the acquisition time. Our protocol (128 projections with a frame length of 10 s) uses a total acquisition time of 11 min on a double-headed gamma camera. The effective dose was estimated to be 3.9 mSv (1.9 mSv for 125 MBq of Technegas [28] and 2 mSv for CT).

Generally, 64 and 96 updates were found optimal for LEHR-125 MBq designs, which is consistent with our earlier result [12]. An even higher number of updates would increase the contrast recovery, but at the same time increase the noise level. An excessively high noise level seemed to diminish the advantage of increased contrast. A soft noise reduction filter with a cutoff frequency of 0.6 to 0.7 cm^−1^ was found optimal.

In the literature on quantitative methods [3,6,7], only use of a high-resolution collimator was found to agree with our results, while the count level and number of iterations varied radically. For qualitative assessment of pulmonary embolism, Palmer et al. [8] showed that a high-resolution collimator, a high number of total counts (5.5 × 10^6^) together with a 128 × 128 matrix of the projections identified the highest number of lesion inserts in a thorax phantom. This agrees well with our results. On the other hand, they used only 16 updates. The contrast recovery achieved with as few as 16 updates would not recover the information that we are interested in.

It was better to use a large kernel size when the lung edge was excluded, but a small kernel size was slightly better when the whole lung was analysed. This can be explained by the following. In a static phantom, the diameter of the lesions was 1 cm and their depth was 50% of the surrounding healthy activity concentration. When the phantom incorporates respiratory motion, the lesions are smoothed, i.e. the lesions become broader and shallower. To this we add the limited resolution of the SPECT system which smoothed the lesions even further. When the lung edge was excluded from the imaged lung, a large kernel encloses more of the lesion gradient, compared to a small kernel, which resulted in higher CV values, i.e. it generated a greater separation between the healthy and COPD distributions. On the other hand, if the edge was included, large kernels at the edges will contain gradients due to lesions *and* lung edge. The lung edge is steeper and equal for both the healthy and COPD distributions while the lesions have shallower gradients. Therefore, a large volume at the periphery of the lung will not contribute to separating the healthy and COPD distributions. The remaining central volume seemed to be too small for the large kernel to gain many high CV values. Therefore, the resulting separation of the imaged distributions will not be greater than for a small kernel. In the case of a small kernel, the remaining central volume will just be somewhat smaller than the reduced lung and therefore only a small increase in separation between the healthy and COPD distributions was found when the lung edge was excluded.

Due to the limited spatial resolution of the SPECT system, the reconstructed activity distribution is blurred which is most clearly seen at the edge of the lung. The voxels involved in the lung edge will therefore result in high CV values, which are not necessarily correlated to the inhomogeneity of the lung activity distribution. Therefore, a reduced lung volume was created with most of this edge effect excluded. Exclusion of the edge layer removes 21% of the original lung volume. This exclusion will be a disadvantage when the peripheral sub-pleural part of the lung is affected.

## Conclusions

The best separation between a simulated healthy and a simulated mild COPD activity distribution using the CV_T_ method was achieved using a total of at least 3.64 × 10^6^ counts in the projections and by employing an LEHR collimator together with a Butterworth power 6 low-pass filter with a cutoff frequency of 0.6 to 0.7 cm^−1^. In the CV_T_ method a kernel approach was used. When the whole lung was analysed, 64 reconstruction updates and a kernel edge length of 1.0 cm gave the best result. For the reduced lung volume, a greater number of updates and a larger kernel size are needed. Suboptimal selections of either cutoff frequency, number of updates and kernel size will reduce the imaging system’s ability to detect mild COPD in the lung phantom, even for 3.64 × 10^6^ counts together with an LEHR collimator.

## Abbreviations

AUC: area under the curve
AUC(CV_T_): area under the curve for CV values greater than CV_T_
CDR: collimator-detector response
COPD: chronic obstructive pulmonary disease
CV: coefficient of variation
CV_T_: CV threshold value
ESSE: effective source scatter estimation
SPECT: single-photon emission computed tomography
